# Outcomes After Salter–Harris II Distal Tibia Fractures in Children

**DOI:** 10.3390/children12010045

**Published:** 2024-12-30

**Authors:** Robert Pearce, Alexander Markes, Toshali Katyal, Jeremy Siu, Ishaan Swarup

**Affiliations:** Department of Orthopaedic Surgery, UCSF Benioff Children’s Hospitals, Oakland, CA 94609, USA; rmo9007@nyp.org (R.P.);

**Keywords:** pediatric trauma, open reduction internal fixation (ORIF), distal tibia fracture, ankle fracture

## Abstract

Background/Objectives: Salter–Harris II (SH-II) distal tibia fractures are the most common physeal ankle fractures in children; however, indications for surgical management remain controversial, and patient-reported outcomes for different management strategies are unknown. The purpose of the current study is to compare differences in clinical and patient-reported outcomes following operative and non-operative management of this injury. Methods: We performed a retrospective cohort study of pediatric patients who were treated at a single institution for SH-II distal tibia fractures between 2013 and 2020. Variables included age, gender, operative versus non-operative treatment, and premature physeal closure (PPC). Patients were also contacted for patient-reported outcome scores (PROs), which included the visual analog scale foot and ankle (VAS-FA) and the PROMIS pediatric mobility instrument obtained at a minimum of 2 years post-injury. Results: Demographic and clinical information was obtained for 46 patients. Our cohort was 52% male with mean age of 11.9 years at injury. At 6 months, the rate of PPC in our cohort was 25%, with no differences between operative and non-operative patients (29% vs. 24%, *p* = 0.80). A total of 15 of the 46 patients provided PROs, with an average follow-up time of 5.1 years (range: 2.9–9.1). VAS-FA and PROMIS pediatric mobility scores were similar between operative and non-operative patients. Conclusions: This pilot study suggests no differences in PROs following operative and non-operative management for SH-II distal tibia fractures; however, future studies with larger cohort sizes and longer follow-up times are needed to further examine these outcomes.

## 1. Introduction

Ankle fractures are among the most common physeal injuries in children, accounting up to 18% of all physeal injuries [1,2,3,4]. These injuries may be limited to the physis (Salter–Harris I) or may involve the distal tibial metaphysis and/or epiphysis (Salter–Harris II-IV). Salter–Harris II fractures are the most common type of physeal fracture of the distal tibia, representing ~40% of all distal tibia fractures in pediatric patients [4,5]. The mean age of injury has been reported to be 12.6 years old, and the fracture is commonly associated with sports [6,7].

Optimal management of this fracture type remains controversial, with considerable variations regarding indications for surgical management, particularly with >3 mm displacement following closed reduction [8]. The most important complication of this injury is premature physeal closure (PPC), which has been estimated to occur in 25–40% of cases [9,10,11,12]. In 2003, Barmada et al. showed that a residual physeal gap >3 mm following reduction was correlated with higher rates of PPC [9]. They argued that PPC rates could be lowered in such cases by performing open reduction with removal of the entrapped periosteum. However, a later study by Russo et al. showed that open reduction and removal of the entrapped periosteum did not reduce PPC rates [7]. Despite extensive study of PPC rates, there is limited data to compare patient-reported outcomes in those treated surgically and non-surgically.

The purpose of this study was to further explore indications for surgical management of SH-II distal tibia fractures by assessing patient-reported outcomes following both operative and non-operative management. We also assessed demographics, radiographic outcomes, and long-term functional outcomes.

## 2. Materials and Methods

The study was reviewed and approved by our institutional review board. We performed a retrospective observational study with prospective follow-up of patients to understand patient-reported outcomes. This study included patients between the ages of 6 and 15 years who underwent treatment for closed Salter–Harris II distal tibia fracture at a single urban center between 2013 and 2020. Participants were identified using the appropriate ICD9 and ICD10 codes: ICD9 823.80, 823.82, 823.90, 823.92, and ICD10 S89.122A. Patients with open fractures and fractures involving the epiphysis were excluded.

**Figure 1:**
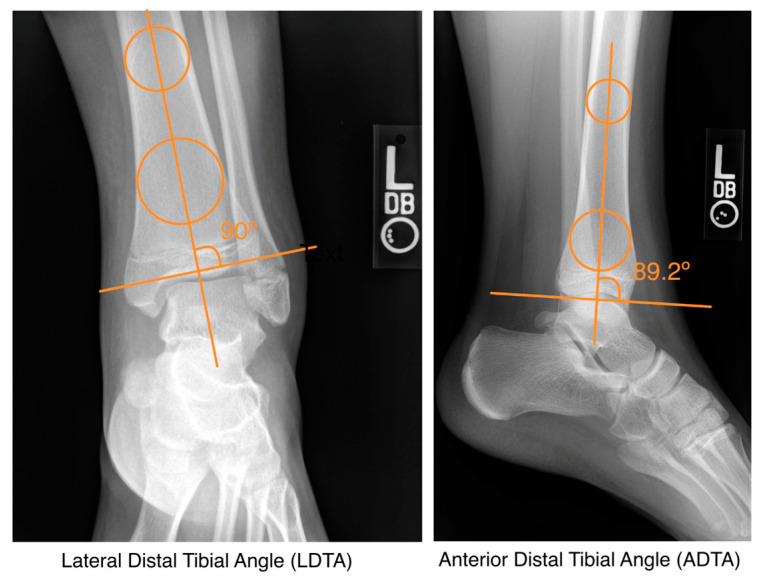
Measurement of lateral distal tibial angle (LDTA) and anterior distal tibial angle (ADTA).

We recorded demographic and clinical factors such as age, sex, race, activity associated with injury, length of follow-up, and management. We performed radiographic reviews of the post-reduction AP, mortise, and lateral X-rays and recorded displacement, anterior distal tibial angle (ADTA), and lateral distal tibial angle (LDTA) (Figure 1). For surgical patients, measurements were taken after closed reduction in the emergency room but before surgical reduction. These parameters were selected to assess the displacement and angulation of the fracture. All radiographs were independently measured by two different reviewers and confirmed by calculating interclass correlation coefficients (ICCs). Any major discrepancies were resolved by a third reviewer. To assess outcomes such as PPC, we identified patients with ≥6 month of radiographic follow-up and evaluated radiographs for PPC. A chart review was also performed to assess the return to sports after operative and non-operative management.

We attempted to contact all patients and families via phone and email to obtain patient-reported outcome measures, and a subset completed the questionnaires. Patient-reported outcomes included the visual analog scale foot and ankle survey (VAS-FA) and the PROMIS pediatric mobility short form. The VAS-FA includes 20 questions which are answered on a visual analog scale and scored out of 100 [13]. The VAS-FA has been validated in adults for a variety of foot- and ankle-specific musculoskeletal conditions [13,14]. The PROMIS pediatric mobility short form is an 8 item form focusing on lower extremity function, which is constructed from a computer-assisted test that has been validated in pediatric lower extremity trauma [15,16]. Each question is scored on a scale of 0–4, and the scores are totaled to calculate a raw score. This raw score is then converted to a T-score based on the reference data, with 50 representing the mean of the US population [17]. Parental consent was obtained in cases in which the patient was younger than 18 years at the time of survey administration. A minimum of 5 attempts were made to contact patients before considering them as lost to follow-up.

The primary outcome measures were rates of PPC and the VAS-FA and PROMIS lower extremity scores. The prospective arm of this study was powered using the VAS-FA. For the power calculation, we assumed a minimally important change (MIC) of 6.8 [18]. No normative data exist on the standard deviation for this specific injury type in children. However, there are data available for children undergoing hindfoot and midfoot osteotomies, which revealed a standard deviation of 6.4 [19]. We calculated a needed sample size of 30 (15 surgical and 15 non-surgical) to achieve 80% power. Any missing data were handled with pairwise deletion. Descriptive statistics were used to summarize the data. Chi-squared and Fisher’s exact tests were used to assess differences between categorical variables. T-tests were used to assess differences between continuous variables. The analysis was conducted in Microsoft Excel (Microsoft, Redmon, WA, USA).

## 3. Results

### 3.1. Patient Demographics, Mechanism of Injury, and Management

**Figure 2:**
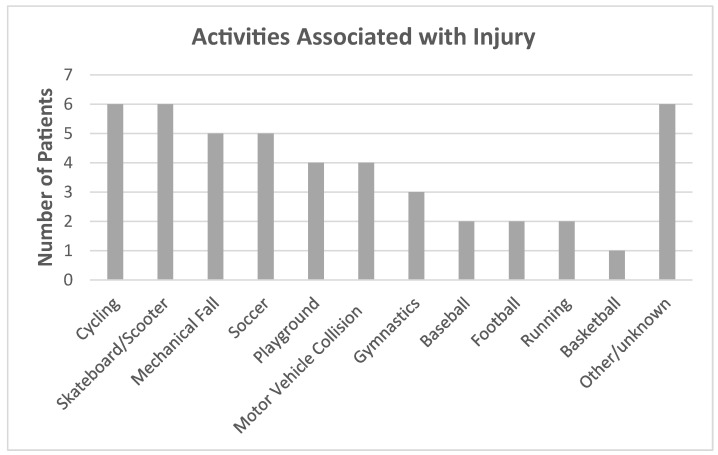
Activities associated with injury.

This study included 46 patients with SH-II distal tibia fractures (Table 1). The mean age at time of injury was 11.9 years (range: 6.5–14.8 years), and 48% of the cohort was female. The female portion of the cohort had a younger mean age than the male portion (10.9 ± 2.0 vs. 12.8 ± 1.3 years, *p* < 0.01). The cohort was 37% Hispanic or Latino, 22% White, and 11% Black. The average clinical follow-up time was 3.0 years with a standard deviation (SD) of 2.4 years. A total of 15 patients completed the surveys, and the average time to follow-up was 5.6 years (range: 4.1–10.2). The majority of injuries were left sided (n = 38, 83%), and half of patients had concomitant fibular fracture. Most injuries were related to sports or recreational activity (n = 31, 67%) (Figure 2).

**Figure 3:**
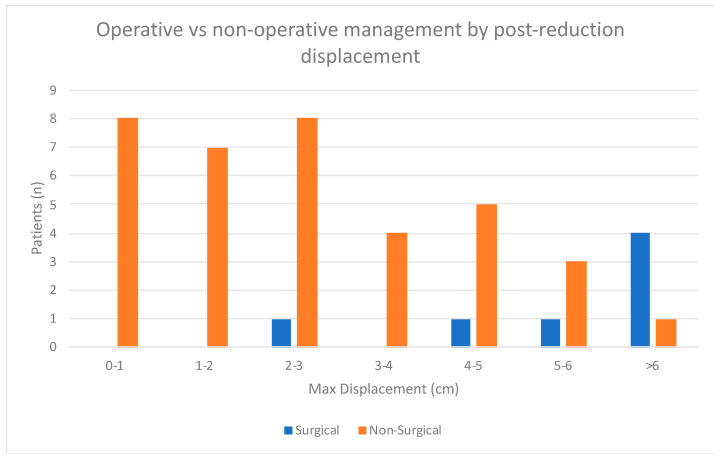
Differences in post-reduction displacement in patients managed operatively and non-operatively.

Approximately 78% of the cohort were managed non-operatively (n = 36), and 22% were managed operatively (n = 10) (Table 2). Of patients managed non-operatively, 15 underwent closed reduction and casting, and 21 underwent casting without reduction. Operative management was comprised of open reduction internal fixation (ORIF) in six patients and closed reduction percutaneous pinning (CRPP) in four patients. Patients managed with surgery were more likely to have concomitant fibular fracture than those treated non-operatively (*p* = 0.03) and had greater post-reduction displacement (8.1 vs. 2.5 mm, *p* = 0.03) (Figure 3). We did not observe significant differences in patient age, sex, race, or mechanism of injury fracture between the two groups (*p* > 0.05). There was improvement in displacement following surgical management. Average displacement improved from 8.1 mm to 1.6 mm (*p* = 0.01). Average LDTA shifted from 82.2° to 84.4° (*p* = 0.49), and average ADTA changed from 88.3° to 84.1° (*p* = 0.24). ICC was 0.91 for displacement, 0.83 for LDTA, and 0.75 for ADTA.

### 3.2. PPC and Return to Sports

**Figure 4:**
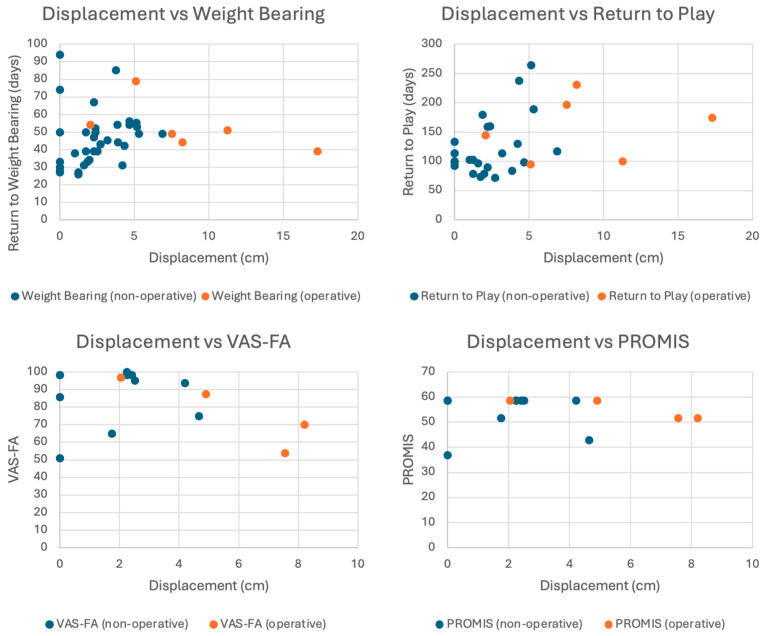
Relationship between displacement, time to weight bearing, time to return to play, VAS-FA, and PROMIS mobility scores.

The average time to weight bearing was 47 days. We did not observe significant differences in the return to weight bearing between operative and non-operative patients (49.3 ± 13.6 vs. 46.0 ± 15.7 days, *p* = 0.57) (Figure 4). The average time to return to sports was 137 days, with no significant differences between operative and non-operative patients (177 ± 77 days vs. 124 ± 51 days, *p* = 0.10). Six months of radiographic follow-up was available for 24 patients. In this group, we observed a PPC rate of 25%, with no difference in rates between patients treated operatively and non-operatively (*p* = 0.79). There was no difference in post-reduction displacement between patients that did or did not develop PPC (3.6 ± 1.4 mm vs. 4.0 ± 4.1, *p* = 0.73).

### 3.3. Patient Reported Outcomes

Patient-reported outcomes were obtained for 15 patients (33% follow-up) (Table 3). The standard deviation was 17.2 for the VAS-FA and 6.7 for the PROMIS pediatric mobility tool across all patients. Average time to this follow-up was 5.1 years (range: 2.9–9.1 years). Five patients were treated operatively, and ten were treated non-operatively. This cohort had a mean post-reduction displacement of 3.05 ± 2.58, LDTA of 87.4 ± 2.8, and ADTA of 87.0 ± 2.5. The mean VAS-FA score was 84.2 out of 100 (range = 50.8–99.6), and the mean PROMIS pediatric mobility T-score was 54.6. The most commonly reported deficiencies in the VAS FA were a higher frequency of pain in physical rest, higher frequency of pain during physical activity, and a higher intensity of pain during physical activity. The most commonly reported deficiency in the PROMIS lower extremity was related to not being able to do sports and exercises that other children of the same age could do. There were no significant differences in VAS-FA and PROMIS scores for operative and non-operative patients (*p* > 0.05, Table 3). When comparing patients with <3 mm and ≥3 mm displacement, VAS FA scores were 87.4 vs. 75.7 (*p* = 0.23), and PROMIS scores were 55.3 vs. 52.6 (*p* = 0.49). Female patients had a younger mean age of injury than male patients (10.9 vs. 12.8 years, *p* < 0.01) and were more likely to report lower PROs (VAS-FA < 80 and PROMIS < 55, *p* = 0.03).

There was concordance between the VAS-FA score and PROMIS tool. In total, 10 patients reported a VAS-FA score > 80, and 5 reported a VAS-FA < 80. All patients with a VAS-FA > 80 reported no deficits with the PROMIS tool (T-score > 55), and all patients with a VAS-FA < 80 reported deficits with the PROMIS tool (T-score < 55).

## 4. Discussion

Salter–Harris II distal tibia fractures are common physeal injuries in children [20]. They are typically acquired through sports injuries and may be managed both operatively and non-operatively. There is considerable inter-surgeon variation on indications for surgical management, suggesting that there may be a clinical equipoise in many cases [8]. Prior studies have shown that PPC, the most important complication of this injury, may occur at similar rates regardless of management strategy [7]. This study builds on prior work by adding a comparison of PROs in patients treated operatively and non-operatively to help surgeons understand the long-term outcomes following both management strategies.

Because our institution does not have any specific protocols or guidelines regarding operative vs. non-operative management, treatment decisions are typically made on a case-by-case basis. Multiple factors are considered, including displacement, angulation, patient symptoms, and patient/family preference. The lack of guidelines and outcome data for this injury type were the main motivations for carrying out this study. The differences between operative and non-operative groups are best summarized in Table 2 and Figure 3.

Our analysis shows that VAS-FA and PROMIS pediatric mobility scores were similar between patients treated operatively and non-operatively, despite the surgical group having significantly greater displacement following initial reduction in the emergency department. The difference in mean VAS-FA of 4.5 was not statistically significant and fell within the reported MIC of 6.8 [18]. Similarly, the difference in mean PROMIS T-scores of 1.6 was not statistically significant and was within estimates of the minimally important difference (MID) of this measure [21,22]. Although limited by a small sample size, these findings suggest that long-term pain and function are similar following both operative and non-operative management, with most respondents noting few, if any, functional deficits. These findings are in concordance with Margalit et al., who assessed functional outcomes in 59 non-operatively treated patients in 2020. They found excellent functional outcomes in patients with SH-II distal tibia fractures and no difference between patients with varying degrees of post-reduction displacement [6]. Our data can also be used to help power a larger study in the future. Based on our observed VAS-FA standard deviation of 17.2, a future study with 80% power, alpha of 0.05, and equal enrollment ratios would require approximately 200 patients (two arms of 100) in order to detect an MIC of 6.8.

Our analysis also supports prior data from Russo et al., who found no difference in PPC rates between patients treated operatively and non-operatively at 6 months follow-up [7,23]. A quarter of our patients had a PPC at 6 months after injury, with no differences between operative and non-operative patients. It is important to note that non-operative patients in our study had less displacement and angulation of their fractures. It is also possible that improving displacement or angulation with operative management [23] may improve long-term outcomes; however, it likely does not affect risk of PPC, which has been shown to be correlated with pronation-abduction injuries and high-grade mechanism [6,12].

Our analysis is also consistent with a meta-analysis comparing PPC rates in SH-I and SH-II distal tibia fractures which also showed no differences based on management [23]. That analysis also noted increased risk of surgical-related complications following operative management, which may contribute to increasing healthcare costs and overall strain on the healthcare system. Given the current clinical equipoise in moderately displaced fractures ~3 mm, surgeons may wish to consider other factors such as patient preference, risk of complications, and increased cost.

Our analysis also revealed that operative patients were weight bearing in a similar time to non-operative patients despite worse fracture characteristics at baseline. The operative patients took longer to return to play, but this difference did not reach statistical significance. The observed difference in return to sports could have been due to the greater displacement in the operatively treated fractures and activity restrictions during the postoperative recovery period. Additional factors such as physical therapy and patient expectations may have affected this finding as well, and standardized protocols such as those seen following ACL reconstruction may be helpful to promote timely patient recovery and return to sports [24,25,26].

This study has several strengths. To our knowledge, this is the first study to report functional outcomes after SH-II distal tibia fractures in pediatric patients. We also used validated outcome measures with an average of 5.1 years follow-up and provided a comprehensive assessment of patient demographics, mechanism of injury, and management. The main limitation of this study was the sample size, which limited the power of the study and also precluded multivariate analyses, which could further elucidate associations between management and outcomes. Our initial power calculation showed that 30 patients would be needed for 80% power and 95% sensitivity, but the observed standard deviation in the PROs was larger than expected, and only 15 patients completed the questionnaires, leading to a possible response bias. The cohort who answered the surveys included both children and adults, leading to challenges selecting the best validated PROs. The VAS-FA is only validated in adults, and the PROMIS pediatric mobility short form has ceiling effects which limit its ability to discern small differences between highly functional individuals. Future studies with larger sample sizes are needed to compare PROs after surgical and nonsurgical management, as well as perform stratified analyses based on fracture displacement and angulation. Additionally, surgeries and follow-up visits were performed by several different surgeons, which could introduce performance biases. However, this study provides a unique, long-term comparison in outcomes between patients with well-aligned distal tibia SH-II Fractures.

## 5. Conclusions

In conclusion, Salter–Harris II distal tibia fractures are common pediatric injuries which often occur in the setting of sports. Long-term outcomes, including patient-reported outcomes, PPC rates, and time to return to activity are similar following operative and non-operative management. Larger, multi-center prospective studies would be helpful to further evaluate functional outcomes and elucidate more clear indications for operative management of this injury based on residual displacement after closed reduction.

## Figures and Tables

**Table 1:** Cohort demographics.

	Overall Cohort (n = 46)
Parameter	n (%)
Age (SD)	11.9 ± 1.9
Female Sex	22 (46)
Race	
White	10 (22)
Black	5 (11)
Hispanic/Latino	18 (39)
Asian	1 (2.2)
Unknown	12 (26)
Sports injury	31 (67)
Left-sided Injury	38 (83)
Concomitant Fibular Fracture	23 (50)

**Table 2:** Surgical outcomes.

	Surgical (n = 10)		Non-Surgical (n = 36)		
Parameter	Mean ± SD	n (%)	Mean ± SD	n (%)	*p*
Age(yrs)	11.8 ± 2.5		11.9 ± 1.7		0.84
Female Sex		3 (30)		19 (53)	0.20
Radiographic Characteristics					
Concomitant fibula fracture		8 (80)		15 (42)	0.03
Postreduction displacement (mm)	8.1 ± 5.0 *		2.5 ± 1.8		0.03
LDTA (°)	82.2 ± 6.3		88.3 ± 2.8		0.07
ADTA (°)	88.3 ± 7.4		86.9 ± 2.7		0.63
Recovery					
Time to Weight Bearing (d)	49.3 ± 13.6		46.0 ± 15.7		0.57
Time to Return to Play **(d)	176.5 ± 77.0		123.5 ± 51.0		0.10
Premature Physeal Closure ^†^		2 (29)		4 (24)	0.80
Patient-Reported outcomes ^‡^					
PROMIS pediatric mobility	55.7 ± 3.8		54.1 ± 7.9		0.59
VAS-FA	81.2 ± 19.3		85.7 ± 16.9		0.67

VAS-FA, Visual Analogue Scale Foot and Anke. * Displacement is after emergency department reduction but before surgical reduction in the operating room. ** n = 8 for the surgical group and n = 24 for the non-surgical group. ^†^ n = 7 for the surgical group and n = 17 for the non-surgical group. ^‡^ n = 5 for the surgical group and n = 10 for the non-surgical group.

**Table 3:** Differences in patient-reported outcomes.

	VAS FA < 80 and PROMIS < 55 (n = 5)		VAS FA > 80 and PROMIS > 55 (n = 10)		
Parameter	Mean ± SD	n (%)	Mean ± SD	n (%)	*p*
Age at injury (yrs)	11.3 ± 2.8		12.9 ± 0.6		0.09
Age at time of Questionaire (yrs)	17.1 ± 2.4		17.7 ± 1.4		0.55
Female Sex		4 (80)		2 (20)	0.03
Operatively managed		2 (40)		3 (30)	0.69
Radiographic Characteristics					
Concomitant fibula fracture		2 (40)		6 (60)	0.46
Postreduction displacement (mm)	4.4 ± 3.6		2.3 ± 1.6		0.26
LDTA (°)	88.4 ± 2.6		86.9 ± 3.0		0.35
ADTA (°)	87.9 ± 1.9		86.5 ± 2.8		0.30

## Data Availability

The raw data supporting the conclusions of this article can be made available by the authors on request. The data are not publicly available due to patient privacy.
